# Supplementary material for: CD28 Autonomous Signaling Up-Regulates C-Myc Expression and Promotes Glycolysis Enabling Inflammatory T Cell Responses in Multiple Sclerosis
Source: Cells. 2019 Jun 11;8(6):575. doi: 10.3390/cells8060575 (PMC6628233; doi:10.3390/cells8060575)
Supplement: Supplementary file 1 [file cells-08-00575-s001.pdf]

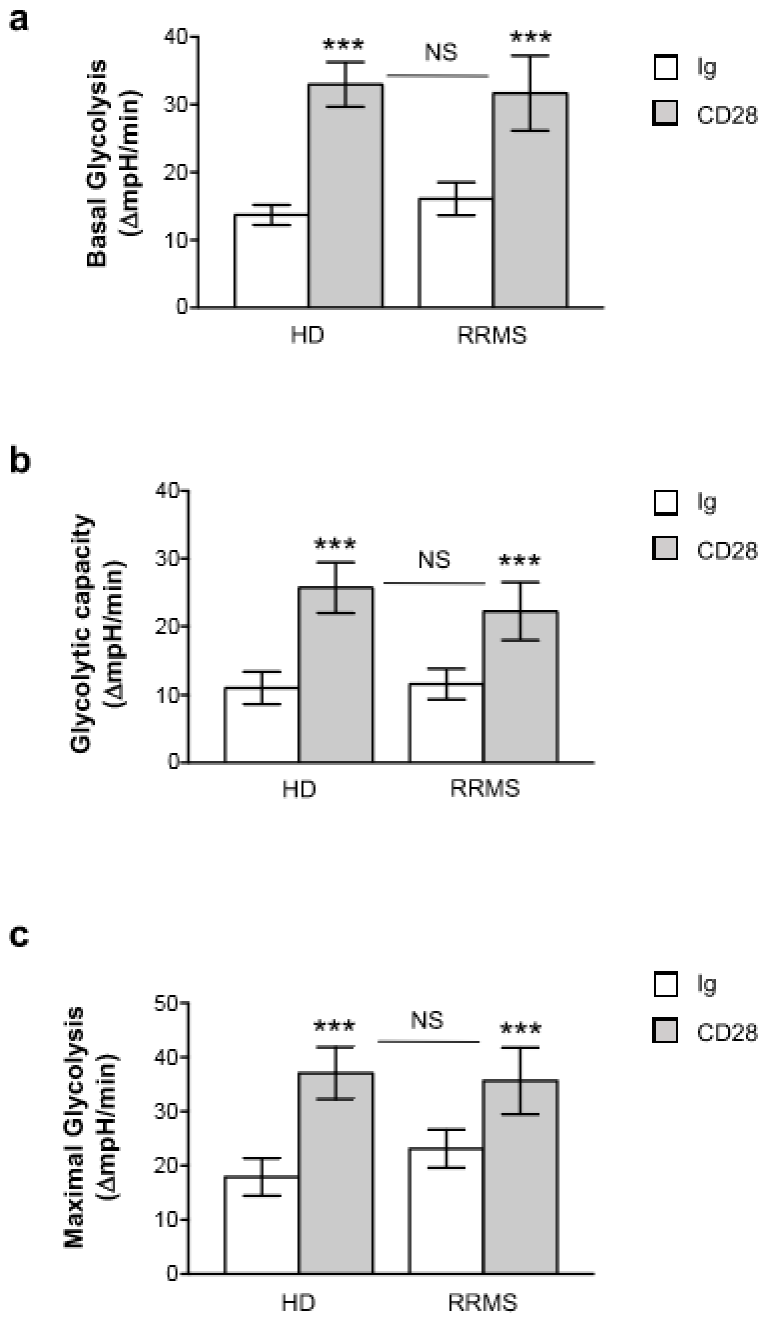

Figure S1. Comparison of CD28-mediated up-regulation of glycolysis between CD4[+] T cells from HD and RRMS. CD4[+] T cells from HD (n = 14) or RRMS patients (n = 14) were stimulated for 18 h with 2 μg mL^{-1} isotype control Ig, or anti-CD28.2 and basal glycolysis after glucose injection (**a**), glycolytic capacity (**b**) and maximal glycolysis (**d**) were calculated from the EACR profiles. Data express mean ± SEM. Significance was calculated by Wilcoxon test. (*) $p < 0.001$. NS = not significant.

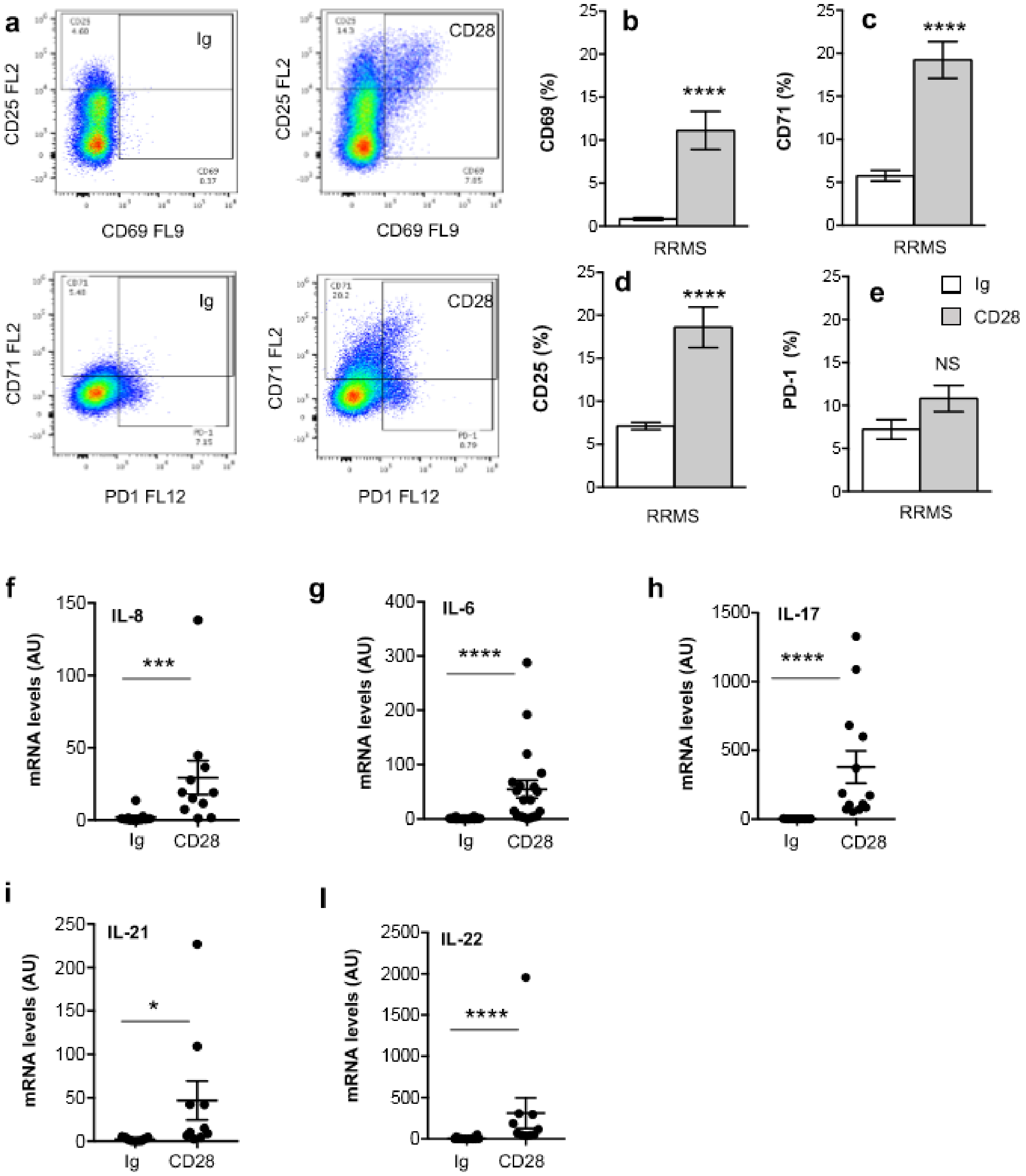

Figure S2. CD28 stimulation up-regulates the expression of surface activation markers and inflammatory cytokines in CD4+ T cells from RRMS patients. (**a–e**) CD4+ T cells from RRMS patients (n = 17) were stimulated for 24 h with control isotype Ig or anti-CD28.2 Abs and the percentage of CD69 (**a, b**), CD71 (**a, c**), CD25 (**a, d**) and PD-1 (**a, e**) on the surface of CD4+ T cells was analysed by multicolour flow cytometry (Cytoflex S, Beckman Coulter). Data express the mean ± SEM and statistical significance was calculated by Student's t test. (**f–l**) CD4+ T cells from RRMS patients were stimulated for 6 h (**f, g, i**) or 24 h (**h, l**) with control isotype Ig or anti-CD28.2 Abs. The mRNA levels of the indicated cytokines were measured by real-time PCR and values, normalized to GAPDH, expressed as arbitrary units (AU). The mean ± SEM are indicated and statistical significance was calculated by Mann-Whitney test. (*) $p < 0.05$, (***) $p < 0.001$, (****) $p < 0.0001$.

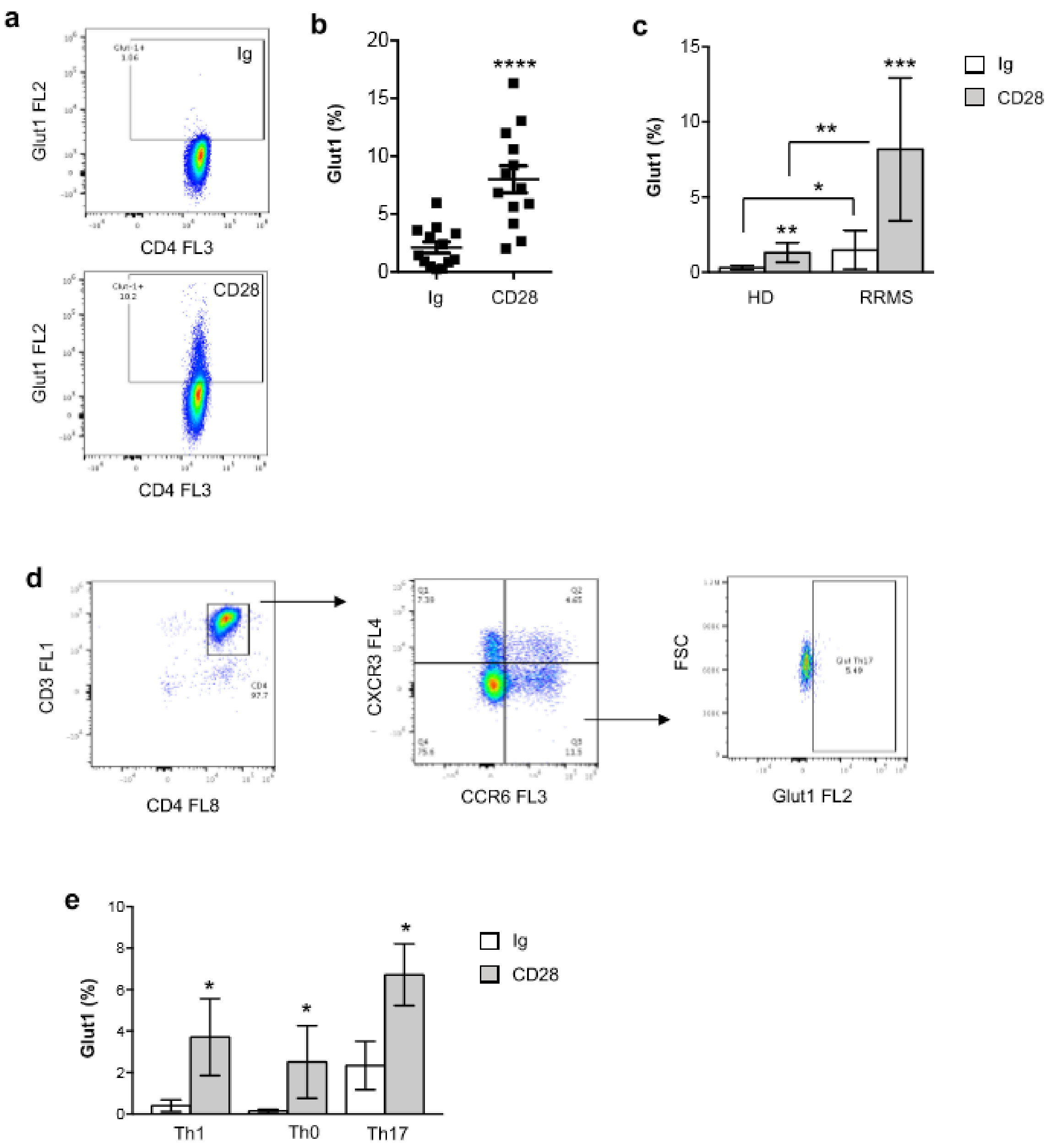

Figure S3. CD28-stimulation up-regulates Glut1 expression in $CD4^+$ T cells from RRMS patients. (**a**–**c**) $CD4^+$ T cells from RRMS patients (*n* = 13) or HD (*n* = 7) were stimulated for 24 h with control isotype Ig or anti-CD28.2 Abs and the expression of Glut1 on the surface of $CD4^+$ T cells was analysed by multicolour flow cytometry. The percentage of Glut1 on total $CD4^+$ T cells from RRMS patients (**b**,**c**) or HD (**c**) was calculated. Data express the mean ± SEM. (**d**) Multicolour flow cytometry analysis of Glut1 expression on $CD4^+CXCR3^+CCR6^-$ Th1-like cells, or $CD4^+CXCR3^-CCR6^+$ Th17-like cells, or $CD4^+CXCR3^-CCR6^-$ Th0-like cells from RRMS patients (*n* = 3) stimulated for 24 h with control isotype Ig or anti-CD28.2 Abs. (**e**) The percentage of Glut1 on Th1, Th0 or Th17 cells was calculated. Data express the mean ± SEM. Statistical significance was calculated by Student's t test. (*) $p < 0.05$, (*) $p < 0.01$, (***) $p < 0.0001$, by Student's t test. NS = not significant.
